# Effects of Operational Parameters on Mg^2+^/Li^+^ Separation Performance in Electrodialysis System

**DOI:** 10.3390/membranes15090260

**Published:** 2025-08-29

**Authors:** Zhijuan Zhao, Jianhua Yang, Dexin Kong, Yunyan Peng, Dong Jin

**Affiliations:** Key Laboratory of Special Equipment Safety and Energy-Saving for State Market Regulation, China Special Equipment Inspection & Research Institute, Beijing 100029, China

**Keywords:** electrodialysis, Mg^2+^/Li^+^ separation performance, high-Mg^2+^/Li^+^-ratio brine

## Abstract

Brine with a high magnesium-to-lithium ratio was separated by electrodialysis equipped with a monovalent cation exchange membrane under differing operational parameters. The ionic concentration variations, separation coefficients, lithium recovery ratio, permselectivity coefficient, and Li^+^ flux were analyzed to evaluate the effect of the initial Li^+^/Mg^2+^ mass concentration ratio, applied voltage, and initial volume ratio between the dilute and concentrated compartments on the separation performance of magnesium and lithium. The results showed that the increase in initial Li^+^/Mg^2+^ concentration ratio significantly increased the separation coefficient, lithium recovery ratio, and Li^+^ flux, demonstrating an improvement in the separation performance since the Li^+^ migration was accelerated when less Mg^2+^ competed with Li^+^. As the applied voltage increased from 10 V to 15 V, the separation coefficient increased, and the lithium recovery ratio and Li^+^ flux increased within 60 min; however, as the applied voltage increased to 20 V, the separation coefficient, the lithium recovery ratio, and the Li^+^ flux did not increase, which indicated that an increase in the applied voltage within the limits would contribute to the separation performance. The increase in the initial volume ratio between the dilute and concentrated compartments decreased the separation coefficient and lithium recovery ratio, indicating that the separation performance had declined.

## 1. Introduction

The global demand for lithium is surging, driven primarily by its critical role in lithium-ion batteries, which dominate the electric vehicle and renewable energy storage markets [1,2]. Projections indicate exponential growth in lithium consumption in the coming years, with the current global demand already exceeding 100,000 tons of lithium carbonate annually [2]. While lithium is traditionally sourced from mineral deposits, the depletion of high-grade ores has intensified the search for alternative resources [3,4]. In this context, lithium extraction from brine has emerged as a promising solution due to its cost efficiency and abundant reserves [5,6,7]. However, a major technical challenge persists: certain brine sources, particularly those with extremely high magnesium-to-lithium mass ratios (MLRs) of up to 365:1, complicate the production of high-purity lithium [5,8]. Consequently, developing selective and economically viable separation technologies for magnesium and lithium has become a priority for the sustainable advancement of the lithium industry.

Membrane-based processes, particularly electrodialysis (ED) and nanofiltration (NF), have gained attention as sustainable alternatives to conventional lithium extraction methods, such as solvent extraction and lime–soda precipitation, which suffer from inefficiency and significant environmental drawbacks [9,10]. Among these, ED stands out as a well-established membrane separation technology, widely applied in seawater desalination, brackish water treatment, and selective ion recovery, including of metal ions [11,12,13,14,15]. This process utilizes an ED stack composed of alternately arranged cation exchange membranes (CEMs) and anion exchange membranes (AEMs), separated by spacers and positioned between an anode (e.g., iridium-coated titanium) and a cathode (e.g., stainless steel) [16]. Under applied electric potential, ions migrate through the membranes based on their charge [17]. Specifically, anions pass through AEMs, while cations pass through CEMs, leading to their separation and concentration. As a result, the ionic concentration increases in the concentrated compartment while decreasing in the dilute compartment, producing two distinct streams: a concentrated solution and a purified dilute solution. This mechanism enables ion recovery and purification, making ED a promising approach to the extraction of ions from brines [18,19].

Conventional cation exchange membranes (CEMs) typically exhibit low selectivity for Li^+^ over Mg^2+^, as the divalent ion is more strongly attracted to the negatively charged membrane matrix despite Li^+^ having higher mobility [20]. To address this limitation, ED systems incorporating monovalent selective ion exchange membranes have been employed for the efficient separation of magnesium and lithium from brines with a high magnesium-to-lithium ratio [21,22]. Moreover, monovalent selective ion exchange membranes typically exhibit high selectivity for lithium ions but demonstrate extremely low selectivity for magnesium ions. As a result, these membranes effectively reduce the magnesium-to-lithium ratio in brine while simultaneously concentrating lithium ions in the concentrated compartment. Studies demonstrate that under an electric field, Li^+^ selectively migrates through the monovalent selective membrane into the concentrated stream, while divalent ions (Mg^2+^, Ca^2+^) are largely retained in the feed solution [23]. The permselectivity between ions of the same charge is quantified by comparing their permeation rates relative to a reference ion (e.g., per equivalent of charge transferred) [24,25,26]. The effect of feed solution characteristics on separation performance has been investigated in previous studies. However, most studies use parameters such as the separation coefficient or permselectivity coefficient to evaluate separation performance, which do not reflect the different changes in it. Therefore, in this work, the effect of the feed solution characteristics on the separation performance was systematically evaluated through multiple parameters.

This study investigates the separation performance of magnesium and lithium from brine with a high magnesium-to-lithium ratio using ED with monovalent cation exchange membranes under various operating conditions. The separation performance was systematically evaluated through multiple parameters: ionic concentration variations, separation coefficient, lithium recovery ratio, permselectivity coefficient, and Li^+^ flux. Key operational parameters, including the initial Li^+^/Mg^2+^ mass concentration ratio, applied voltage, and volume ratio between the diluted and concentrated compartments, were examined to determine their effects on separation performance. Through the comprehensive analysis of the parameters for separation performance, optimal ED operating conditions were identified for the effective separation of magnesium and lithium.

## 2. Materials and Methods

### 2.1. Experimental Setup and Materials

The electrodialysis setup is mainly composed of a membrane stack, dilute solution tank, concentrated solution tank, electrode solution tank, tubes, pumps and direct-current power supply. The membrane stack is composed of ten pairs of cation exchange membranes (CEMs) and anion exchange membranes (AEMs), separated by spacers and positioned between an anode and a cathode. The CEM is the commercial monovalent selectivity cation exchange membrane CIMS, and the AEM is the commercial standard anion exchange membrane AMD; they were purchased from Hangzhou Lanran Technology Co., Ltd. (Hangzhou, China). The size of the ion exchange membrane is 19.5 cm × 7.5 cm, and the effective membrane area of each membrane is 55 cm^2^. The thickness of both CIMS and AMD is 0.10 mm, and the thickness of the spacers is 0.9 mm. The anode and cathode are both iridium-coated titanium electrodes. Anhydrous sodium sulfate (Na_2_SO_4_, AR, 99%), lithium chloride (LiCl, AR, 99%), and magnesium chloride hexahydrate (MgCl_2_·6H_2_O, AR) were purchased from Aladdin Industrial Co., Ltd. (Shanghai, China).

### 2.2. Electrodialysis Experiment

**Figure 1:**
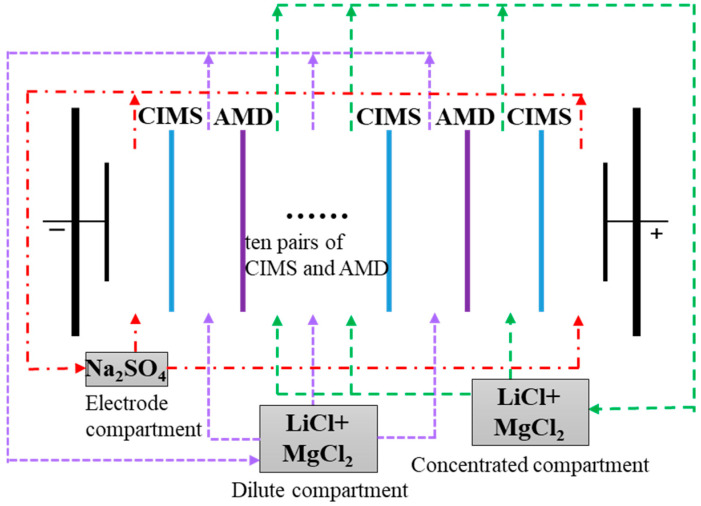
The schematic diagram of the electrodialysis experiment.

The schematic diagram of the electrodialysis experiment is shown in Figure 1; the electrodialysis experiment is performed at 25 °C. The dilute compartment is filled with the mixed solution of LiCl and MgCl_2_, and the mass concentration of Li^+^ and Mg^2+^ is 200 and 8000 mg/L, respectively. The concentrated compartment is filled with the same solution as the dilute compartment. The volume of both the dilute compartment and the concentrated compartment is 1 L. The electrode compartment is filled with 1 L of 0.3 mol/L Na_2_SO_4_. The dilute solution and concentrated solution are circulated into the dilute compartment and concentrated compartment, respectively, driven by a peristaltic pump with a flow rate of 120 L/h. The process is performed at a constant applied voltage. The electrodialysis experiment is performed for 120 min, and a sample is taken every 20 min. The ion concentration (i.e., Li^+^ and Mg^2+^) of each sample is measured using inductively coupled plasma optical emission spectrometry (ICP-OES, Agilent 5110, Santa Clara, CA, USA). The Mg^2+^ concentration in the mixed solution is changed to 2000 mg/L, 4000 mg/L, and 16,000 mg/L, respectively, to investigate the effect of the initial Li^+^/Mg^2+^ mass concentration ratio on the separation performance. The applied voltage is changed to 10 V, 15 V, and 20 V, respectively, to investigate the effect of the applied voltage on the separation performance. The volume of the dilute compartment and concentrated compartment is changed to 1 L, 0.5 L, and 0.8 L, 1 L, respectively, to investigate the effect of the initial volume ratio between the dilute and concentrated compartments on separation performance.

### 2.3. Characterization of Separation Performance

#### 2.3.1. Separation Coefficient

The separation coefficient of magnesium and lithium refers to the ratio of Mg^2+^ to Li^+^ at time *t* over the initial ratio of Mg^2+^ to Li^+^ in the dilute compartment. The larger the separation coefficient, the better the separation performance of magnesium and lithium.

$$F_{Mg-Li}=\frac{c_{{Mg}^{2+}}^{t}/c_{{Li}^{+}}^{t}}{c_{{Mg}^{2+}}^{0}/c_{{Li}^{+}}^{0}}$$

where $c_{{Mg}^{2+}}^{t}$ and $c_{{Li}^{+}}^{t}$ represent the concentration (mg/L) of magnesium and lithium in the dilute compartment at time *t*, respectively. $c_{{Mg}^{2+}}^{0}$ and $c_{{Li}^{+}}^{0}$ represent the initial concentration (mg/L) of magnesium and lithium in the dilute compartment, respectively.

#### 2.3.2. Lithium Recovery Ratio

The lithium recovery ratio is calculated as follows:

$$R_{{Li}^{+}}=\frac{V_{ct}(c_{ct}-c_{c0})}{V_{d0}c_{do}}$$

where $c_{ct}$ is the lithium concentration (mg/L) in the concentrated compartment at time t. $c_{c0}$ and $c_{do}$ are the initial lithium concentration (mg/L) in the concentrated compartment and dilute compartment, respectively. $V_{d0}$ is the initial solution volume (L) in the dilute compartment. $c_{ct}$ and $V_{ct}$ are the lithium concentration (mg/L) and solution volume (L) in the concentrated compartment at time *t*.

#### 2.3.3. Permselectivity Coefficient

The permselectivity coefficient of lithium and magnesium refers to the ratio of Li^+^ to Mg^2+^ in the concentrated compartment at time *t* over the initial ratio of Li^+^ to Mg^2+^ in the dilute compartment.

$$P_{Li/Mg}=\frac{c_{{Li}^{+}}^{t^{\prime }}/c_{{Mg}^{2+}}^{t^{\prime }}}{c_{{Li}^{+}}^{0}/c_{{Mg}^{2+}}^{0}}$$

where $c_{{Li}^{+}}^{t^{\prime }}$ and $c_{{Mg}^{2+}}^{t^{\prime }}$ represent the concentration of lithium and magnesium in the concentrated compartment, respectively. $c_{{Li}^{+}}^{0}$ and $c_{{Mg}^{2+}}^{0}$ represent the initial concentration (mg/L) of lithium and magnesium in the dilute compartment, respectively.

#### 2.3.4. Ionic Flux

Ionic flux is the rate of transfer of specific ions through the membrane per unit of time and area, which can be calculated as follows:

$$J=\frac{V_{t}(c_{t}-c_{0})}{A\times t}$$

where $c_{0}$ is the initial ion concentration (mg/L) in the concentrated compartment. $c_{t}$ and $V_{t}$ are the ion concentration (mg/L) and solution volume (L) in the concentrated compartment at time *t*. *A* is the effective membrane area (cm^2^), and *t* is the running time (s).

## 3. Results and Discussion

The ED process was carried out with different initial Li^+^/Mg^2+^ mass concentration ratios (1:10, 1:20, 1:40, and 1:80), applied voltages (10 V, 15 V, and 20 V) and initial volume ratios between the dilute and concentrated compartments (1:0.5, 1:1, and 0.8:1) to investigate the effect of operational parameters on the separation performance of magnesium and lithium.

### 3.1. Effect of Operational Parameters on the Ionic Concentration Variation

**Figure 2:**
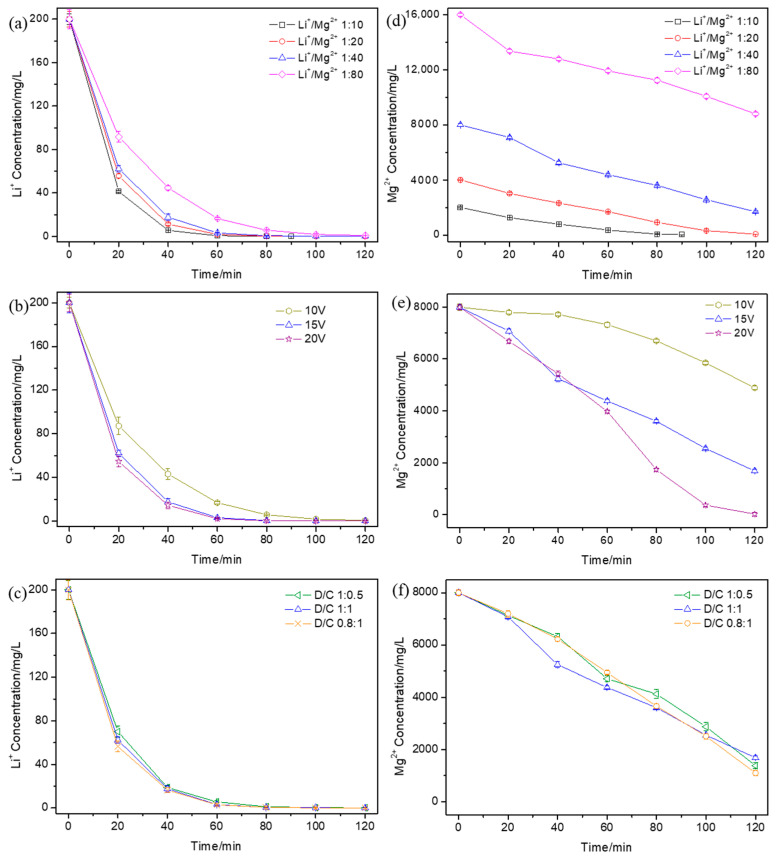
The variation in (**a**–**c**) Li^+^ concentration and (**d**–**f**) Mg^2+^ concentration in the dilute compartment with time during electrodialysis under different operational parameters: (**a**,**d**) with different initial Li^+^/Mg^2+^ concentration ratios when the applied voltage was 15 V and the initial volume ratio between the dilute and concentrated compartments was 1:1, (**b**,**e**) at different applied voltages when the initial Li^+^/Mg^2+^ concentration ratio was 1:40 and the initial volume ratio between the dilute and concentrated compartments was 1:1, and (**c**,**f**) with different initial volume ratios between the dilute and concentrated compartments when the applied voltage was 15 V and the initial Li^+^/Mg^2+^ concentration ratio was 1:40.

The concentration of Li^+^ and Mg^2+^ in the dilute compartment during the ED process under different operational parameters was measured and is shown in Figure 2. Over time, the concentrations of Li^+^ and Mg^2+^ both decreased to different degrees. The concentrations of Li^+^ and Mg^2+^ during ED with different initial Li^+^/Mg^2+^ mass concentration ratios are shown in Figure 2a and Figure 2d, respectively. With the decrease in the initial Li^+^/Mg^2+^ concentration ratio, the decline rate of Li^+^ concentration slowed down; however, the Li^+^ concentration was nearly 0 after 120 min. The decline rate of the Mg^2+^ concentration showed almost no change with the change in the initial Li^+^/Mg^2+^ concentration ratio. It can be seen that the decrease in the initial Li^+^/Mg^2+^ concentration ratio mainly exhibited an influence on the migration of Li^+^ since, with the decrease in the initial Li^+^/Mg^2+^ concentration ratio, more Mg^2+^ would compete with Li^+^ and hinder Li^+^ migration, which made the decline rate of the Li^+^ concentration slow down. However, the increase in the amount of Mg^2+^ di not change its migration rate, which led to no change in the decline rate of the Mg^2+^ concentration. The concentrations of Li^+^ and Mg^2+^ during ED at different applied voltages are shown in Figure 2b and Figure 2e, respectively. With the applied voltage increase from 10 V to 15 V, the decline rate of the Li^+^ and Mg^2+^ concentration was accelerated; with the applied voltage increase from 15 V to 20 V, the decline rate of Li^+^ showed almost no change and the decline rate of Mg^2+^ showed almost no change up until 60 min and then acceleration after 60 min. The concentrations of Li^+^ and Mg^2+^ during ED with different initial volume ratios between the dilute and concentrated compartments are shown in Figure 2c and Figure 2f, respectively. With the increase in the initial volume ratio between the dilute and concentrated compartments, the decline rate of the Li^+^ concentration slowed down up until 40 min and then showed almost no change. The decline rate of the Mg^2+^ concentration exhibited no differences. This was because that the concentration gradient between the dilute compartment and concentrated compartment increased with the increase in the initial volume ratio; hence, the Li^+^ migration resistance caused by the concentration gradient increased and less Li^+^ migrated into the concentrated compartment.

### 3.2. Effect of Operational Parameters on Separation Coefficient

**Figure 3:**
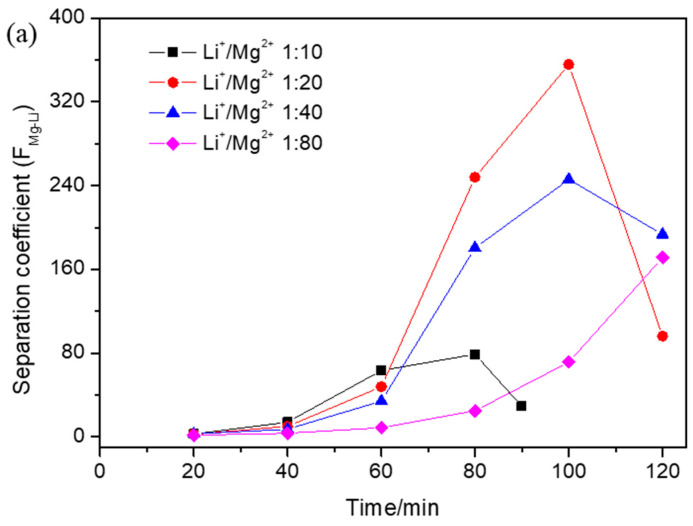

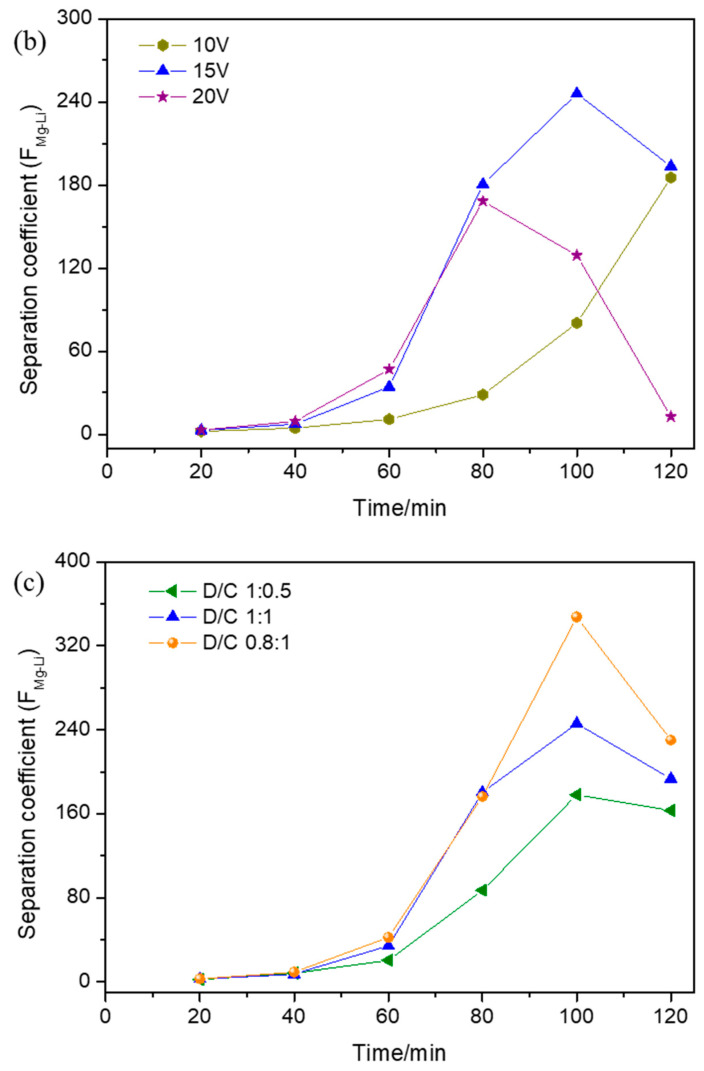
The effect of the (**a**) initial Li^+^/Mg^2+^ concentration ratio, (**b**) applied voltage, and (**c**) initial volume ratio between the dilute and concentrated compartments on the separation coefficient (F_Mg-Li_) during electrodialysis when (**a**) the applied voltage was 15 V and the initial volume ratio between the dilute and concentrated compartments was 1:1, (**b**) the initial Li^+^/Mg^2+^ concentration ratio was 1:40 and the initial volume ratio between the dilute and concentrated compartments was 1:1, and (**c**) the applied voltage was 15 V and the initial Li^+^/Mg^2+^ concentration ratio was 1:40.

The separation coefficient of magnesium and lithium under different operational parameters was calculated and is shown in Figure 3. Over time, the separation coefficient of magnesium and lithium obviously increased and then declined at the end of the ED process since the Li^+^ concentration was very low and the migration of Mg^2+^ was the main process. The separation coefficient of magnesium and lithium under different initial Li^+^/Mg^2+^ mass concentration ratios is shown in Figure 3a. As the initial Li^+^/Mg^2+^ concentration ratio decreased, the separation coefficient simultaneously decreased up until 60 min. After that, the separation coefficient increased slightly and then decreased when the Li^+^/Mg^2+^ ratio was 1:10; however, the separation coefficient increased obviously and then decreased when the Li^+^/Mg^2+^ ratio was 1:20 and 1:40, and the separation coefficient increased slightly and then increased obviously when the Li^+^/Mg^2+^ ratio was 1:80. With the initial Li^+^/Mg^2+^ concentration ratio decrease from 1:10 to 1:20, the highest separation coefficient increased, and with the initial Li^+^/Mg^2+^ concentration ratio decrease from 1:20 to 1:80, the highest separation coefficient decreased. With the decrease in the initial Li^+^/Mg^2+^ concentration ratio, the Li^+^ migration rate decreased due to there being a higher Mg^2+^ obstacle, which made the separation coefficient decrease. However, when the initial Li^+^/Mg^2+^ concentration ratio was 1:10, both Li^+^ and Mg^2+^ concentrations were low after 60 min, which made the separation coefficient low. Therefore, the highest separation coefficient was achieved when the initial Li^+^/Mg^2+^ concentration ratio was 1:20. The separation coefficients of magnesium and lithium at different applied voltages are shown in Figure 3b. Over time, the separation coefficients of magnesium and lithium obviously increased and then declined at the end of the ED process, and the separation coefficient decreased earlier as the applied voltage increased. With the increase in the applied voltage, the separation coefficient simultaneously increased up until 70 min, and then the separation coefficient at 20 V was near that at 15 V, which was higher obviously than that at 10 V. However, the separation coefficient reached its peak when the applied voltage was 15 V. With the applied voltage increase from 10 V to 15 V, the Li^+^ migration rate accelerated and the separation coefficient improved. When the applied voltage increased to 20 V, the Li^+^ migration rate showed almost no change and Mg^2+^ migration rate accelerated, which made the separation coefficient decline. Hence, the increase in the applied voltage within limits would contribute to the Mg^2+^/Li^+^ separation [19]. The separation coefficients of magnesium and lithium with different initial volume ratios between the dilute and concentrated compartments are shown in Figure 3c. Over time, the separation coefficient of magnesium and lithium obviously increased and then declined at the end of the ED process. With the increase in the initial volume ratio between the dilute and concentrated compartments, the separation coefficient simultaneously decreased. This was because the Li^+^ concentration increased and the Mg^2+^ concentration showed almost no change with the increase in the initial volume ratio between the dilute and concentrated compartments, and the separation coefficient decreased.

### 3.3. Effect of Operational Parameters on Lithium Recovery Ratio

**Figure 4:**
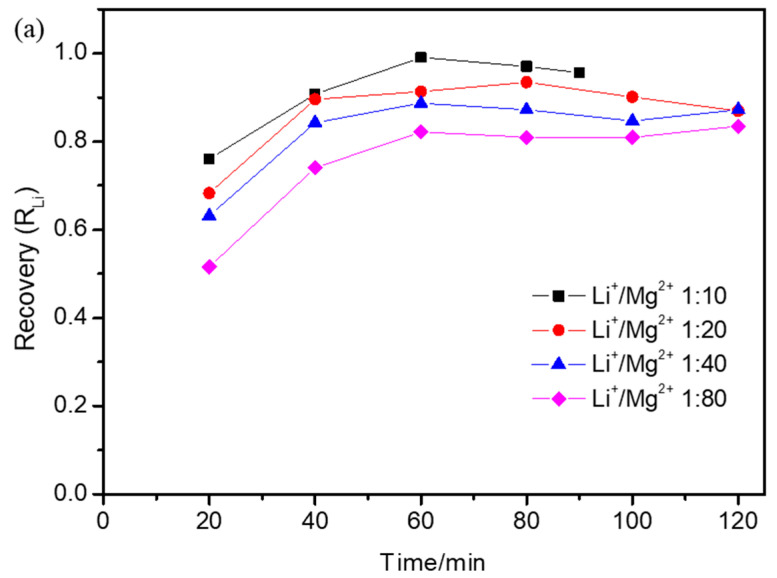

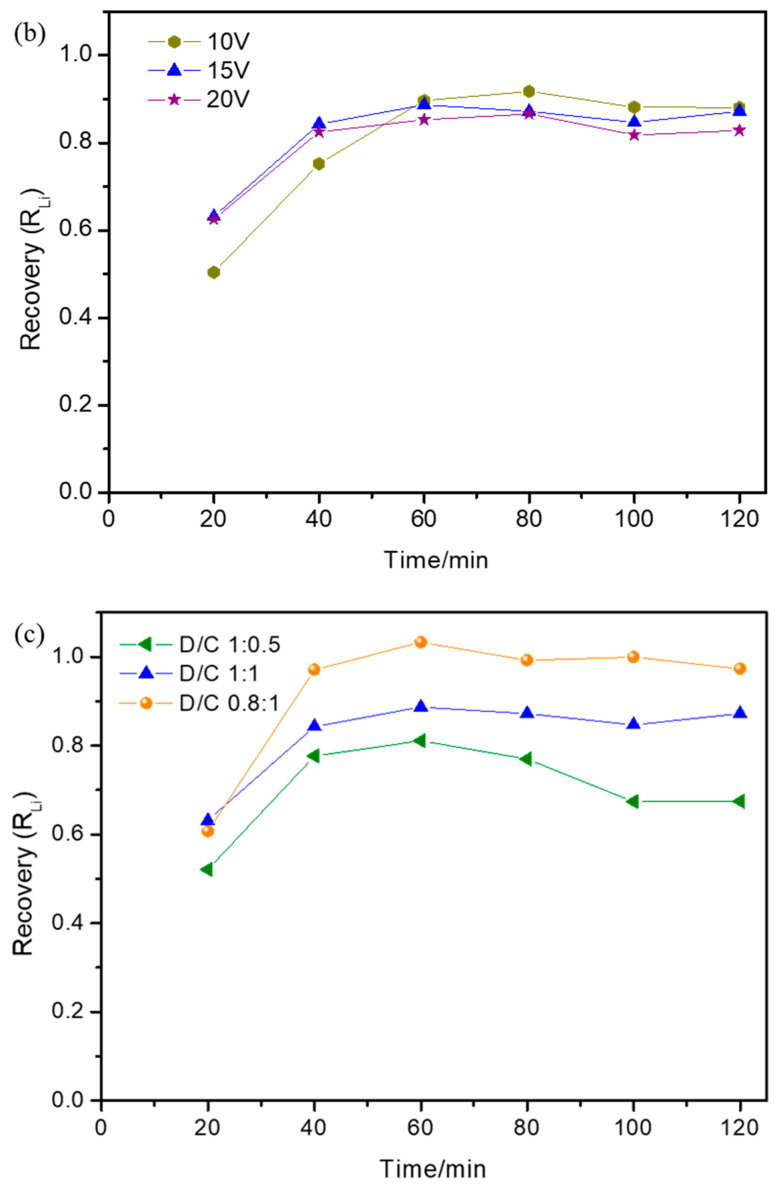
The effect of the (**a**) initial Li^+^/Mg^2+^ concentration ratio, (**b**) applied voltage, and (**c**) initial volume ratio between the dilute and concentrated compartments on lithium recovery (R_Li_) during electrodialysis when (**a**) the applied voltage was 15 V and the initial volume ratio between the dilute and concentrated compartments was 1:1, (**b**) the initial Li^+^/Mg^2+^ concentration ratio was 1:40 and the initial volume ratio between the dilute and concentrated compartments was 1:1, (**c**) the applied voltage was 15 V and the initial Li^+^/Mg^2+^ concentration ratio was 1:40.

The lithium recovery ratio under different operational parameters was calculated and is shown in Figure 4. Over time, the lithium recovery ratio firstly increased and then slightly decreased. Figure 4a showed the lithium recovery ratio with different initial Li^+^/Mg^2+^ mass concentration ratios. The lithium recovery ratio increased obviously up until 60 min and then decreased slightly. This was because Li^+^ migrated quickly from the dilute compartment to the concentrated compartment up until 60 min, and then the Li^+^ concentration in the dilute compartment was near to 0 and the Li^+^ concentration in the concentrated compartment was almost unchanged; however, the volume of the concentrated compartment decreased slightly, which made the lithium recovery ratio increase obviously up until 60 min and then decrease slightly. With the decrease in the initial Li^+^/Mg^2+^ concentration ratio, the lithium recovery ratio simultaneously decreased. This was because Li^+^ concentration in the concentrated compartment decreased due to there being a higher Mg^2+^ obstacle with the decrease in the initial Li^+^/Mg^2+^ concentration ratio, which also indicated a decline in the separation performance, consistent with the results given above. Figure 4b shows the lithium recovery ratio at different applied voltages. The lithium recovery ratio increased obviously up until 60 min and then showed almost no change. The lithium recovery ratio at 15 V was a little higher than that at 20 V, and both were higher than that at 10 V before 60 min and lower than that at 10 V after 60 min. Up until 60 min, Li^+^ migrated quickly from the dilute compartment to the concentrated compartment; with the applied voltage increase from 10 V to 15 V, the Li^+^ migration rate increased obviously, making the lithium recovery ratio at 15 V higher than that at 10 V; with the applied voltage increase to 20 V, the Li^+^ migration rate showed almost no change. The back diffusion made the Li^+^ concentration in the concentrated compartment decrease slightly; thus, the lithium recovery ratio was slightly lower than that at 15 V. After 60 min, the Li^+^ concentration in the dilute compartment was near to 0 and the back diffusion made the Li^+^ concentration decrease slightly in the concentrated compartment; moreover, with the applied voltage increase, the decline rate of the Li^+^ concentration was accelerated, which made the lithium recovery ratio decrease. Figure 4c shows the lithium recovery ratio with different initial volume ratios between the dilute and concentrated compartments. The lithium recovery ratio increased obviously up until 40 min and then decreased slightly. With the increase in the initial volume ratio between the dilute and concentrated compartments, the lithium recovery ratio simultaneously decreased. After 40 min, most Li^+^ had migrated to the concentrated compartment and the Li^+^ concentration with different initial volume ratios between the dilute and concentrated compartments showed no obvious difference. The lithium recovery ratio was mainly related to the differences in volume ratios between the dilute and concentrated compartments.

### 3.4. Effect of Operational Parameters on Permselectivity Coefficient

**Figure 5:**
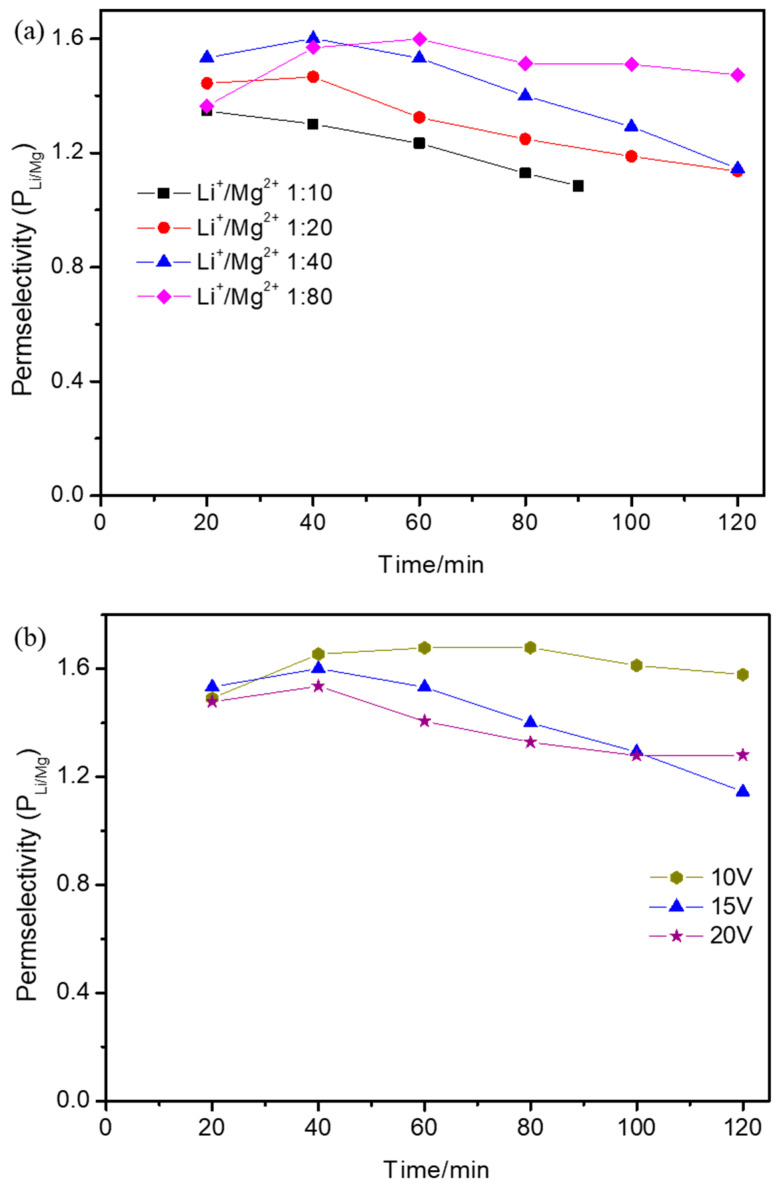

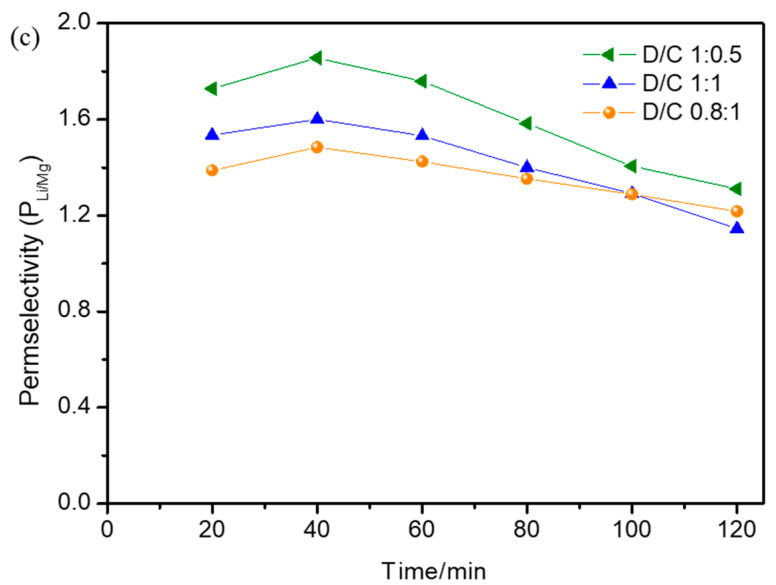
The effect of the (**a**) initial Li^+^/Mg^2+^ concentration ratio, (**b**) applied voltage, and (**c**) initial volume ratio between the dilute and concentrated compartments on the permselectivity (P_Li/Mg_) during electrodialysis when (**a**) the applied voltage was 15 V and the initial volume ratio between the dilute and concentrated compartments was 1:1, (**b**) the initial Li^+^/Mg^2+^ concentration ratio was 1:40 and the initial volume ratio between the dilute and concentrated compartments was 1:1, and (**c**) the applied voltage was 15 V and the initial Li^+^/Mg^2+^ concentration ratio was 1:40.

The permselectivity coefficient under different operational parameters was calculated and is shown in Figure 5. Over time, the permselectivity coefficient increased up until 40 min and then decreased, except for when the initial Li^+^/Mg^2+^ ratio was 1:10, in which case the permselectivity coefficient decreased with time. This is because, up until 40 min, Li^+^ migrated to the concentrated compartment prior to Mg^2+^, which made the permselectivity coefficient increase. After 40 min, most Li^+^ had migrated to the concentrated compartment and the Li^+^ concentration showed almost no change, but the Mg^2+^ concentration still increased obviously in the concentrated compartment, which made the permselectivity coefficient decrease. However, when the initial Li^+^/Mg^2+^ ratio was 1:10, most Li^+^ migrated to the concentrated compartment much earlier than 20 min, and after that, the Li^+^ concentration showed almost no change but the Mg^2+^ concentration still increased; hence, the permselectivity coefficient decreased with time. Figure 5a shows the permselectivity coefficient with different initial Li^+^/Mg^2+^ concentration ratios. As the initial Li^+^/Mg^2+^ concentration ratio decreased from 1:10 to 1:80, the highest permselectivity coefficient increased. With the decrease in the initial Li^+^/Mg^2+^ concentration ratio, more Mg^2+^ competed with Li^+^ and the Li^+^ concentration in the concentrated compartment decreased at the same time, but the Mg^2+^ concentration showed almost no change. The decrease rate of the Li^+^/Mg^2+^ concentration in the concentrated compartment was lower than that of the initial Li^+^/Mg^2+^ concentration ratio, which made the permselectivity coefficient increase with the decrease in the initial Li^+^/Mg^2+^ concentration ratio. Figure 5b shows the permselectivity coefficient at different applied voltages. With the increase in the applied voltage, the permselectivity coefficient simultaneously decreased. This was because the concentrations of Li^+^ and Mg^2+^ in the concentrated compartment both increased with the increase in the applied voltage and the increased rate of the Mg^2+^ concentration was much higher than that of the Li^+^ concentration. Figure 5c shows the permselectivity coefficients with different initial volume ratios between the dilute and concentrated compartments. With the increase in the initial volume ratio between the dilute and concentrated compartments, the permselectivity coefficient simultaneously increased. The concentrations of Li^+^ and Mg^2+^ in the concentrated compartment both increased with the increase in the initial volume ratio between the dilute and concentrated compartments, and the increase rate of the Li^+^ concentration was much higher than that of the Mg^2+^ concentration, which made the permselectivity coefficient increase.

### 3.5. Effect of Operational Parameters on Li^+^ Flux

**Figure 6:**
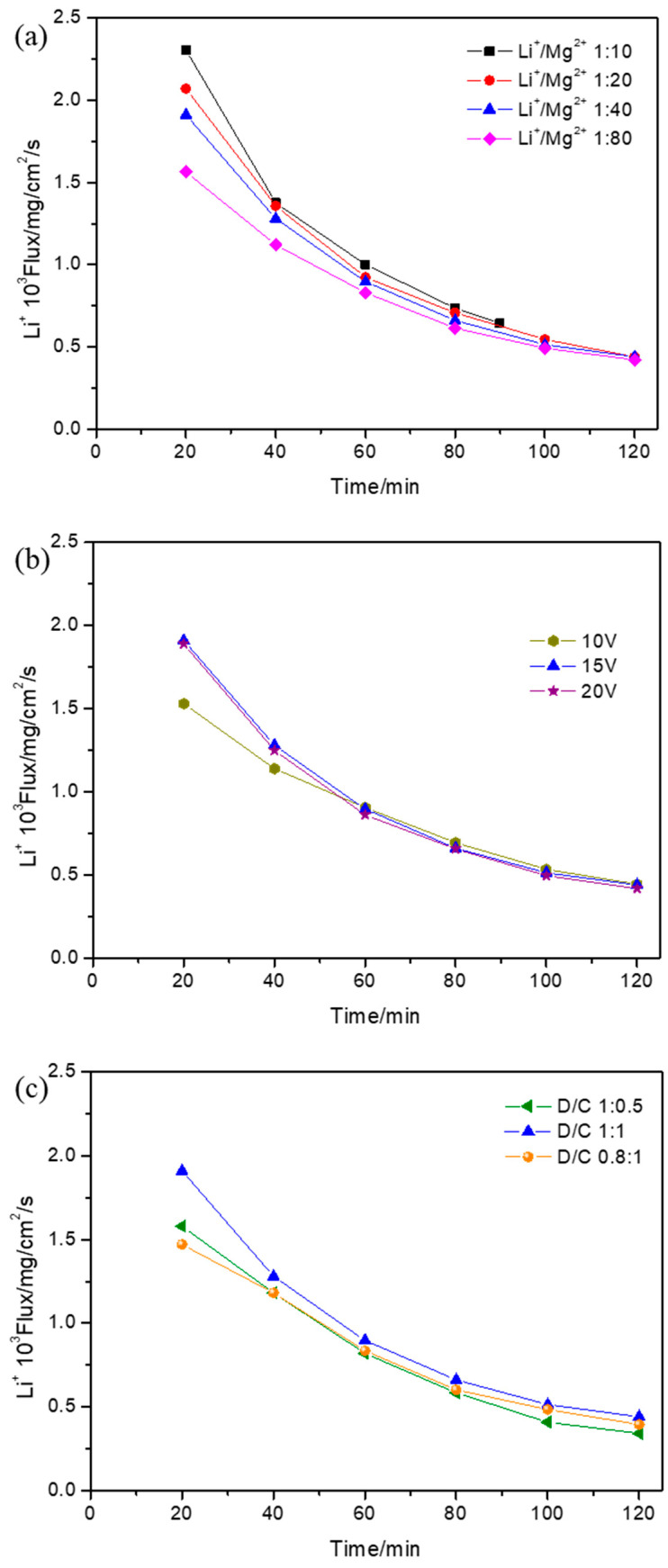
The effect of the (**a**) initial Li^+^/Mg^2+^ concentration ratio, (**b**) applied voltage, and (**c**) initial volume ratio between the dilute and concentrated compartments on the lithium flux (J_Li_) during electrodialysis when (**a**) the applied voltage was 15 V and the initial volume ratio between the dilute and concentrated compartments was 1:1, (**b**) the initial Li^+^/Mg^2+^ concentration ratio was 1:40 and the initial volume ratio between the dilute and concentrated compartments was 1:1, and (**c**) the applied voltage was 15 V and the initial Li^+^/Mg^2+^ concentration ratio was 1:40.

The Li^+^ flux during the ED process under different operational parameters was calculated and is shown in Figure 6. Over time, the Li^+^ flux declined under different operational parameters. The Li^+^ flux during ED with different initial Li^+^/Mg^2+^ concentration ratios is shown in Figure 6a. With the decrease in the initial Li^+^/Mg^2+^ concentration ratio, the Li^+^ flux simultaneously declined. The flux differences with different initial Li^+^/Mg^2+^ concentration ratios became smaller as time went on, and the Li^+^ flux was the same at 120 min because the Li^+^ concentration in the concentrated compartment decreased due to there being a greater Mg^2+^ obstacle with the decrease in the initial Li^+^/Mg^2+^ concentration ratio. The Li^+^ flux during ED at different applied voltages is shown in Figure 6b. The Li^+^ flux at 10 V was lower than that at 15 V up until 60 min and then was the same as that at 15 V. Moreover, at this time, the Li^+^ fluxes at 15 V and 20 V exhibited no differences. With the applied voltage increasing from 10 V to 15 V, the Li^+^ migration rate increased up until 60 min, and after that, most Li^+^ had migrated to the concentrated compartment and the Li^+^ concentration was almost the same. With the applied voltage increasing from 15 V to 20 V, the Li^+^ migration rate showed almost no change, which was consistent with the Li^+^ concentration change in the dilute compartment. The Li^+^ flux during ED with different initial volume ratios between the dilute and concentrated compartments is shown in Figure 6c. The Li^+^ flux firstly increased and then decreased with the increase in the initial volume ratio between the dilute and concentrated compartments. Since the Li^+^ concentration in the concentrated compartment increased with the increase in the initial volume ratio between the dilute and concentrated compartments, the Li^+^ flux with the initial volume ratio of 1:1 was higher than that with 0.8:1. However, the volume of the concentrated compartment decreased sharply under the initial volume ratio of 1:0.5, which meant that the Li^+^ flux under the initial volume ratio of 1:0.5 was lower than that under 1:1.

## 4. Conclusions

The effect of operational parameters including the initial Li^+^/Mg^2+^ concentration ratio, applied voltage, and initial volume ratio between the dilute and concentrated compartments on the separation performance of magnesium and lithium during the ED process was studied in this work. The ionic concentration variations, separation coefficient, lithium recovery ratio, permselectivity coefficient, and Li^+^ flux were measured and calculated to characterize the separation performance. With the increase in the initial Li^+^/Mg^2+^ concentration ratio, the separation coefficient, lithium recovery ratio, and Li^+^ flux increased, which indicated that the separation performance had improved because of the Mg^2+^ obstacle being smaller. With the applied voltage increasing from 10 V to 15 V, the separation coefficient improved and the lithium recovery ratio and Li^+^ flux increased until 60 min; with the applied voltage increasing to 20 V, the separation coefficient, lithium recovery ratio, and Li^+^ flux did not increase. This indicated that an increase in the applied voltage improved the separation performance, while excessive applied voltage was not beneficial to the separation performance. With the increase in the initial volume ratio between the dilute and concentrated compartments, the separation coefficient and lithium recovery ratio decreased, which indicated that the separation performance declined because of the decrease in the Li^+^ migration rate. These results could provide a reference for the choice of operational parameters for the separation of magnesium and lithium using ED.

## Data Availability

The original contributions presented in this study are included in the article. Further inquiries can be directed to the corresponding authors.
